# Effectiveness of Relactation Supportive Program in Sustaining Exclusive Breastfeeding: A Prospective Observational Study

**DOI:** 10.7759/cureus.75413

**Published:** 2024-12-09

**Authors:** Shetanshu Srivastava, Nidhika Pandey, Arvind Kumar Shukla

**Affiliations:** 1 Department of Pediatrics, Dr. Ram Manohar Lohia Institute of Medical Sciences, Lucknow, IND; 2 Department of Pediatrics, Rama Medical College and Research Centre, Kanpur, IND; 3 Department of Pediatrics, Maharaja Suheldev Autonomous State Medical College, Bahraich, IND

**Keywords:** breastfeeding, breastfeeding promotion, infant nutrition, relactation, relactation supportive program

## Abstract

Introduction: Relactation is the process of re-establishing breastfeeding after stopping or after a period of little breastfeeding. The study aimed to assess the Relactation Supportive Program (RSP)'s efficacy in sustaining breastfeeding and to determine the impact of RSP on breastfeeding initiation, timing, and correlation with the lactation gap.

Methods: A prospective observational study was done with 60 infant-mother dyads, aged seven days to 14 weeks who stopped breastfeeding for 6-28 days or never breastfed. Mothers in the RSP group received hands-on guidance on techniques like supplemental feeding methods and nipple stimulation, alongside psychological and emotional support. In the Routine Counseling (RC) group, mothers received counseling about the benefits of breastfeeding, breast milk expression, correct attachment, and motivation for breastfeeding.

Results: In the RSP group, 21 infants achieved and sustained exclusive breastfeeding by six months (p=0.003), compared to 10 in the RC group. RSP initiated lactation earlier (10.04±4.02 days, p=0.002) than the RC group (13.43±3.59 days). Relactation duration was inversely correlated with the lactation gap.

Conclusion: RSP significantly promoted earlier and sustained relactation compared to RC alone.

## Introduction

Breastfeeding has a biological and emotional impact on the health and well-being of both infants and mothers [1]. The first two years of an infant's life constitute a critical period for growth and development. Consequently, the global health policy advocates for exclusive breastfeeding for the first six months, followed by continued breastfeeding along with safe, appropriate, and adequate complementary feeding [2]. Breastfeeding has a pivotal role in preventing malnutrition and infections [3,4]. Delayed initiation of breastfeeding and inappropriate feeding practices can exacerbate the risk of undernutrition in infants and young children [5]. Despite the advantages of breastfeeding, the timing of breastfeeding initiation and the introduction of complementary feeding vary considerably across different cultural, demographic, and societal contexts [6,7].

The breastfeeding rate is 41.6% in India according to the National Family Health Survey, 2015-16 (NFHS-4), and while approximately 51% of infants receive exclusive breast milk for the first two to three months of life, this rate decreases to 28% by the age of four to five months [8]. Additionally, early initiation of breastfeeding, within one hour after birth, remains low at 24.5% in India [9]. The implications of low compliance with exclusive breastfeeding for the first six months of an infant's life are substantial. As per the World Health Organization's child growth standards, 39% of children under six months in India are underweight [9]. Furthermore, infants who do not receive exclusive breastfeeding for the first six months face a higher risk of gastrointestinal infections, respiratory illnesses, morbidity, and even mortality [2,10]. These risks extend to atopic eczema, allergies, asthma, type II diabetes, leukemia, and obesity in later life [11-13]. Given the crucial role of exclusive breastfeeding in both short-term and long-term child health, it is imperative to take proactive steps to promote exclusive breastfeeding. 

Relactation is the process of re-establishing breastfeeding after a period of cessation or very limited breastfeeding [14]. It can be either partial or complete relactation and offers a potentially lifesaving measure. Mothers who relactate can produce enough milk for their infants [15]; hence, relactation can be successfully achieved with adequate support [16]. We thus planned this study to evaluate the impact of a Relactation Supportive Program (RSP) on breastfeeding initiation, timing, and duration and compare its effectiveness with routine counseling.

## Materials and methods

This was a prospective observational study, conducted at the Department of Pediatrics of Era's Lucknow Medical College and Hospital, Lucknow, India, over a period of 24 months from February 14, 2017, to February 14, 2019. Ethical clearance was obtained from the Institutional Ethical Committee of Era's Lucknow Medical College and Hospital (approval number: ELMC/R_CELL/EC/2017/63). Written informed consent was obtained from all participating mothers prior to their enrollment in the study.

Inclusion and exclusion criteria

Inclusion criteria were infants aged 7-14 weeks and mothers who had either never initiated breastfeeding or had discontinued breastfeeding for a period of 6-28 days. Exclusion criteria were infants requiring hospitalization for any medical condition, infants with congenital malformations, and mothers with significant medical conditions, such as diabetes mellitus or hypertension.

Sample size calculation

A sample size of 60 mother-infant dyads was calculated based on anticipated effect sizes from prior study [17] on time of relactation commencement. A power analysis was conducted to ensure the sample size was sufficient to detect statistically meaningful differences between the groups. The formula, n=2.(Zα/2+Zβ)^2^⋅σ^2^, was employed, where n is the sample size required per group, σ is the standard deviation of the outcome (time to lactation commencement, based on the prior study), Zα/2 is the Z-score corresponding to the desired significance level (1.96 for a 95% confidence level), and Zβ is the Z-score corresponding to the desired power (80% power).

Study population and group allocation

Thus, a total of 60 mother-infant dyads were enrolled in the study. Participants were allocated into two groups of 30 based on their treatment choices during routine clinical consultations. (i) RSP Group: Mothers in this group received counseling addressing breastfeeding issues such as lactation gaps, reduced milk production, and breast conditions. Additionally, the mothers received hands-on guidance on techniques like supplemental sucking technique, which is fixing the feeding tubes on the mothers' breasts and encouraging infants to suckle while receiving supplementary feeding. The drip-and-drop method was also employed, which is gradual supplementation via drip feeding to stimulate breastfeeding. Nipple stimulation was done to encourage frequent placement of the infant at the breast to stimulate milk production. Counseling was adapted to individual needs, done twice a day or more. (ii) RC Group: Mothers in this group were counseled about the benefits of breastfeeding, breast milk expression, correct attachment, and motivation for breastfeeding without the specific sessions of RSP as mentioned above.

Data collection and follow-up

Baseline demographic data were collected for all mother-infant dyads, including maternal age, education, occupation, parity, mode of delivery, lactation gap, and infant characteristics such as gender, gestational age, and prior neonatal intensive care unit (NICU) admission. Both groups were followed up at one week, four weeks, and six months. During each follow-up, the following outcomes were assessed: the commencement of lactation, breastfeeding frequency during day and night achievement of exclusive breastfeeding, and anthropometric parameters of infants, including weight, length, and head circumference. In the RSP group, counselling was reinforced at all three follow-up visits to ensure adherence and resolve any emerging challenges.

Data analysis

Data were entered into IBM SPSS Statistics for Windows, Version 19.0 (Released 2010; IBM Corp., Armonk, New York, United States) for statistical analysis. Continuous variables were summarized using means and standard deviations, while categorical variables (e.g., education level, parity) were presented as frequencies and percentages. An independent samples t-test was used to compare continuous variables between the RSP and RC groups. Categorical variables were analyzed using the chi-square test; a p-value < 0.05 was considered statistically significant

## Results

Out of the 60 cases enrolled in the study, 30 (50%) were assigned to each group, out of which two in each group were lost to follow-up. The demographic profiles of mothers in both groups were comparable, as shown in Table 1. The mean age of mothers in the RSP group was 25.46±3.41 years, and in the RC group was 26.00±3.48 years. No significant differences were observed between the two groups in terms of age, parity, education, working conditions, place of residence, or mode of delivery (p>0.05). The mean lactation gap in the RSP group was 10.79±4.67 days, and in the RC group, was10.04±3.31 days, which was comparable.

**Table 1:** Demographic profile of mother and infant dyad (N=56) LSCS: lower segment caesarean section; NICU: neonatal intensive care unit; RSP: Relactation Supportive Program; RC: routine counseling

Maternal Characteristics	RSP Group (n=28)	RC Group (n=28)	Chi-square test (X^2^)	p-value
	n (%)	n (%)		
Age in years				
<25 years	11 (39.3)	9 (32.1)	NA	0.577
>25 years	17 (60.7)	19 (67.9)		
Mean±SD	25.46±3.41	26.00±3.48		
Education				
Illiterate	7 (25.0)	7 (25.0)	0.650	0.885
High school	8 (28.6)	8 (28.6)		
Intermediate	9 (32.1)	7 (25.0)		
Graduate	4 (14.3)	6 (21.4)		
Occupation				
Employed	18 (64.3)	19 (67.9)	0.080	0.778
Unemployed	10 (35.7)	9 (32.1)		
Parity				
Primi para	15 (53.6)	16 (57.1)	0.072	0.788
Multi para	13 (46.4)	12 (42.9)		
Mode of delivery				
LSCS	8 (28.6)	14 (50.0)	2.695	0.101
Normal	20 (71.4)	14 (50.0)		
Lactation gap				
<12 days	19 (67.9)	19 (67.9)	0	1
>12 days	9 (32.1)	9 (32.1)		
Mean±SD (Range) in days	10.79+4.67 (6-21)	10.04+3.31 (6-16)		
Infant Characteristics				
Post-natal age				
<6 weeks	3 (10.7)	6 (21.4)	1.58	0.45
6-12 weeks	23 (67.9)	19 (82.1)		
>12 weeks	2 (7.1)	3 (10.7)		
Mean±SD	9.55±2.84	8.90±3.21		
Gender				
Male	13 (46.4)	19 (67.9)	2.625	0.105
Female	15 (53.6)	9 (32.1)		
Pre-term/Term				
Pre-term	10(35.7)	6(21.4)	1.400	0.237
Term	18(65.3)	22(78.6)		
Pre-lacteal feed				
Yes	7 (25)	8 (28.6)	0.091	0.763
No	21 (75)	20 (71.4)		
NICU admission				
Yes	7 (25.0)	2 (7.1)	3.310	0.069
No	21 (75.0)	26 (92.9)		

Infant baseline characteristics like age and gender were comparable in both groups. The mean age was 9.55±2.84 days in the RSP group vs 8.90±3.21 days in the RC group. Out of the total infants in the RSP group, 10 (35.7%) were pre-term, seven (25%) had NICU admission, and seven (25%) received pre-lacteal feeds. In the RC group, six (21.4%) were preterms, eight (28.6%) infants received pre-lacteal feeds, and two (7.1%) had NICU admission. There was no significant difference between the groups.

Commencement of lactation was significantly earlier in the RSP group (10.04±4.02 days) compared to the RC group (13.43±3.59 days, p=0.002). The lactation gap also affected the commencement of relactation in both groups, as shown in Table 2.

**Table 2:** Comparison of time taken for commencement of lactation Unpaired t-test, *Significant RSP: Relactation Supportive Program; RC: routine counseling

Commencement of lactation	RSP Group (N=28)	RC Group (N=28)	t-test	p-value
Time taken (days)	10.04±4.02	13.43±3.59	3.328	0.002*

Out of 28 infants, 21 (75%) achieved exclusive breastfeeding in the RSP group, which was statistically significant (p=0.003), compared to 10 out of 28 infants (35.7%) in the RC group, as shown in Table 3.

**Table 3:** Comparison of two groups with respect to exclusive breastfeeding status *Significant RSP: Relactation Supportive Program; RC: routine counseling

Exclusive breastfeeding till 6 months	RSP Group (n=28)	RC Group (n=28)	Chi-square test(X^2^)	p-value
	n (%)	n (%)		
Yes	21 (75)	10 (35.7)	8.743	0.003*
No	7 (25)	18 (64.3)		

Regarding day and night feeding frequencies, by the first week, mothers in the RSP group exhibited significantly higher feeding frequencies during both the day (p=0.001) and night (p=0.02). This trend continued through the subsequent follow-ups at four weeks and six months, with the RSP group consistently showing significantly higher feeding frequencies (p=0.001 for both daytime and nighttime), as shown in Table 4.

**Table 4:** Effect of RSP vs RC on day and night feeds on follow-up Unpaired t-test, *Significant RSP: Relactation Supportive Program; RC: routine counseling

Frequency of feeding	RSP group (N=28), mean±SD	RC group (N=28), mean±SD	t-test	p-value
Day time				
1 week	5.50±1.87	3.79±1.19	4.082	0.001*
4 weeks	7.82±1.98	5.50±1.97	4.395	0.001*
6 months	9.82±1.82	8.18±1.82	3.372	0.001*
Night time				
1 week	6.29±2.22	5.04±1.85	2.289	0.02*
4 weeks	8.68±2.48	6.64±1.54	3.698	0.001*
6 months	10.00±1.78	8.46±1.37	3.628	0.001*

The anthropometric parameters of the infants, including weight, length, and head circumference, were measured at enrollment and at different follow-up intervals (one week, four weeks, and six months) to assess growth outcomes in the RSP and RC groups. The baseline parameters were comparable between the two groups, and no statistically significant differences were observed in weight, length, or head circumference at any follow-up interval, as shown in Table 5.

**Table 5:** Comparison of anthropometric parameters of the infants between two groups at enrolment and at different follow-up intervals Unpaired t-test RSP: Relactation Supportive Program; RC: routine counseling

Anthropometric parameters /Time periods	RSP group (N=28), mean±SD	RC group (N=28), mean±SD	t-test	p-value
Weight in kilogram				
At enrolment	3.73 ±0.77	3.59±0.81	0.663	0.522
1 week	4.30±0.81	4.09±0.80	0.976	0.339
4 weeks	5.25±0.87	5.15±0.82	0.443	0.673
6 months	7.01±1.03	6.88±1.00	0.443	0.619
Length in centimeter				
At enrolment	51.45±2.37	50.48±2.37	1.532	0.131
1 week	51.60±2.40	50.83±2.27	1.233	0.226
4 weeks	55.99±2.47	55.23±2.01	1.263	0.210
6 months	61.29±1.46	60.46±1.73	1.940	0.060
Head circumference				
At enrolment	33.90±1.39	33.79±1.31	0.305	0.768
1 week	34.43±1.50	34.23±1.39	0.518	0.594
4 weeks	36.37±1.69	36.62±1.74	0.545	0.588
6 months	39.30±1.39	39.49±1.39	0.511	0.605

## Discussion

Our findings demonstrate that the RSP was effective in promoting earlier commencement of lactation as in other studies [18-23] and achieving exclusive breastfeeding compared to routine breastfeeding counseling alone. By the six-month follow-up, 75% of infants in the RSP group had successfully achieved and maintained exclusive breastfeeding, compared to 35.7% in the RC group. This difference was statistically significant (p=0.003). Additionally, the mean time for lactation commencement was notably shorter in the RSP group (10.04±4.02 days) compared to the RC group (13.43±3.59 days) (p=0.002), underscoring the advantages of the structured interventions provided in the RSP.

The demographic characteristics of mothers, such as age, were consistent with previous studies [17,24], indicating that women aged 20-30 years are more likely to respond positively to RSPs. Education, often considered a key influencing factor, did not differ significantly between the groups, suggesting that it did not act as a confounder in this study. However, 68.5% of mothers in the study were working, highlighting occupational commitments as a potential contributor to breastfeeding discontinuation, as noted in other studies [17,25]. Breast milk expression was a part of breastfeeding counseling, and it helped them keep up with their work commitments. Primiparous women also faced greater challenges with breastfeeding, aligning with findings from earlier research [26]. These observations underscore the importance of tailoring relactation interventions to address specific challenges faced by these groups.

The lactation gap in this study ranged from six to 21 days, with 67.9% of participants experiencing a gap of less than 12 days. The mean lactation gap was comparable between the RSP group (10.79±4.67 days) and the RC group (10.04±3.31 days). Previous studies have highlighted the critical role of shorter lactation gaps in achieving relactation success. For example, De et al. [20] reported that 56.8% of cases had gaps exceeding 16 days, while Tomar et al. [26] found gaps of more than 14 days in 58% of cases. Consistent with these findings, our study observed a significant relationship between shorter lactation gaps and reduced time to relactation initiation, highlighting the importance of early intervention.

Infant characteristics, such as gestational age and pre-lacteal feeding, did not differ significantly between the groups. However, 66.7% of infants in the RSP group were full-term and aged between six and 12 weeks. This aligns with other studies [26-28], which included infants under three months to facilitate effective follow-up until six months of age. While prematurity has been identified as a factor contributing to lactation failure in other research [29], we did not observe it in our study. This could be because we took preterm infants who did not have any medical complications.

The success of relactation commencement was influenced by a combination of factors, including the structured techniques provided in the RSP. Techniques such as supplemental sucking and the "drip-and-drop" method, along with frequent nipple stimulation, proved to be effective in achieving favorable outcomes. Unlike some studies that used pharmacological interventions, our approach relied solely on non-pharmacological methods, further emphasizing the practicality and accessibility of the RSP. Including a group that received RC allowed us to understand the RSP's effectiveness.

A continuous increase in breastfeeding frequency during both daytime and nighttime was observed in the RSP group. This reflects the higher success rate of complete relactation in this group, leading to sustained milk production over time. The ability of the RSP to promote exclusive breastfeeding is particularly important for the infant's long-term growth, development, and immunity, reinforcing its critical role in improving health outcomes.

Despite the promising results, our study has certain limitations. It was conducted at a single center with a relatively small sample size, which may limit the generalizability of the findings. Future multi-center studies with larger sample sizes are recommended to validate these results further and explore the applicability of the RSP in diverse populations.

## Conclusions

Relactation support can play a crucial role in re-establishing and sustaining successful breastfeeding and promoting infant health. Our study highlights the importance of early intervention and support for mothers facing breastfeeding issues with relatively shorter lactation gaps. This study underscores the critical role of structured RSP in promoting exclusive breastfeeding, which is vital for infant growth, and development. Comprehensive support programs should be implemented promptly to improve breastfeeding outcomes.
